# Obesity modulates the immune macroenvironment associated with breast cancer development

**DOI:** 10.1371/journal.pone.0266827

**Published:** 2022-04-26

**Authors:** Aleida Núñez-Ruiz, Flor Sánchez-Brena, Cynthia López-Pacheco, Naray A. Acevedo-Domínguez, Gloria Soldevila

**Affiliations:** 1 Departamento de Inmunología, Instituto de Investigaciones Biomédicas, UNAM, México City, México; 2 Laboratorio Nacional de Citometría de Flujo, Instituto de Investigaciones Biomédicas, UNAM, México City, México; The University of Burdwan, INDIA

## Abstract

Growing evidence demonstrates a strong correlation between obesity and an increased risk of breast cancer, although the mechanisms involved have not been completely elucidated. Some reports have described a crosstalk between adipocytes, cancer cells, and immune cells within the tumor microenvironment, however, it is currently unknown whether obesity can promote tumor growth by inducing systemic alterations of the immune cell homeostasis in peripheral lymphoid organs and adipose tissue. Here, we used the E0771 breast cancer cell line in a mouse model of diet-induced obesity to analyze the immune subpopulations present in the tumors, visceral adipose tissue (VAT), and spleen of lean and obese mice. Our results showed a significant reduction in the frequency of infiltrating CD8^+^ T cells and a decreased M1/M2 macrophage ratio, indicative of the compromised anti-tumoral immune response reported in obesity. Despite not finding differences in the percentage or numbers of intratumoral Tregs, phenotypic analysis showed that they were enriched in CD39^+^, PD-1^+^ and CCR8^+^ cells, compared to the draining lymph nodes, confirming the highly immunosuppressive profile of infiltrating Tregs reported in established tumors. Analysis of peripheral T lymphocytes showed that tumor development in obese mice was associated to a significant increase in the percentage of peripheral Tregs, which supports the systemic immunosuppressive effect caused by the tumor. Interestingly, evaluation of immune subpopulations in the VAT showed that the characteristic increase in the M1/M2 macrophage ratio reported in obesity, was completely reversed in tumor-bearing mice, resembling the M2-polarized profile found in the microenvironment of the growing tumor. Importantly, VAT Tregs, which are commonly decreased in obese mice, were significantly increased in the presence of breast tumors and displayed significantly higher levels of Foxp3, indicating a regulatory feedback mechanism triggered by tumor growth. Altogether, our results identify a complex reciprocal relationship between adipocytes, immune cells, and the tumor, which may modulate the immune macroenvironment that promotes breast cancer development in obesity.

## Introduction

Breast cancer (BrCa) is the most prevalent cancer in women, with an estimated 2.26 million new cases in 2019 and nearly 685,000 deaths worldwide (GLOBOCAN, 2020). It has been reported that around 39% of the world adult population is obese (BMI ≥ 30 kg/m^2^) or overweight (BMI 25.0–29.9 kg/m^2^). Indeed, obesity has become an emerging epidemic strongly associated with several metabolic disorders, including diabetes, fatty liver, cardiovascular diseases [1], and cancer [2]. Notably, obesity has been strongly correlated with poor prognosis, diminished relapse-free survival, higher mortality, and increased risk of metastasis in BrCa patients [3–5].

Obesity is a low-grade chronic inflammatory disease, associated with an excess of nutrient storage in adipose tissue (AT), which results in local and systemic immune alterations [6]. Several groups have described the mechanisms by which AT resident immune subpopulations are affected by obesity. Resident M2 macrophages play an important anti-inflammatory role in AT, contributing to the systemic metabolic homeostasis [7–9]. In obesity, nutrient excess causes the hypertrophy and hyperplasia of adipocytes, leading to local hypoxia and a pro-inflammatory milieu, which favors the induction of necrotic foci [10, 11]. This leads to the polarization of M2 adipose tissue macrophages (ATM) into the M1 phenotype, which induce the formation of “crown-like structures” (CLS), considered a distinctive feature of inflammation in AT [12, 13].

Other types of immune cells have been reported to be altered in fat AT. In homeostasis, AT is abundantly infiltrated by anti-inflammatory cytokine-producing subpopulations such as Tregs, T helper type 2 (T_H_2) cells, ILC2, and eosinophils. In obesity, these subpopulations are displaced by pro-inflammatory cytokine-producing cells like T helper type 1 (T_H_1) cells, CD8+ cytotoxic T cells, and NK cells, which are major producers of IFNγ [10, 14]. These changes are mainly attributed to the overproduction of leptin within the AT, which precedes both the accumulation and polarization of M1 macrophages [10].

Regarding the mechanisms underlying the increased susceptibility of BrCa in obese patients [11, 15], fat adipocytes contribute to tumor development by the production of pro-inflammatory adipokines, including IL-6, IL-1β, TNF-α, and leptin [16–19]. In addition, free fatty acids (FFA) can promote the proliferation of cancer cells [20, 21]. Reciprocally, tumor cells can promote the de-differentiation of adipocytes and their conversion into cancer associated adipocytes (CAAs), which in turn produce pro-inflammatory cytokines, proteases (e.g., PAI-1 [22], MMP11 [23], and FFA). This inflammatory milieu enhances tumor invasiveness and metastasis to secondary organs [19–21, 24, 25].

Alongside adipocytes, stromal cells and endothelial cells, immune cells are a prominent component of the tumor microenvironment (TME). The profile of the infiltrating immune cells evolves with the development of the tumoral mass [26]. In the early stages, innate and adaptive immune cell subpopulations with cytotoxic activity (such as CD8^+^ T lymphocytes or NK cells) are recruited to the tumor [27]. As the tumor progresses, cancer cells shape the tumor infiltrate profile by secreting chemotactic factors such as CCL2 (which recruits myeloid cells), and cytokines like TGF-β and IL-10 (which promote an immunosuppressive milieu), favoring the continued growth of the tumor [28]. In this context, Tregs, M2-like TAM, and myeloid-derived suppressor cells (MDSC) are highly enriched within the established tumor, correlating with an impaired anti-tumoral response and poor prognosis in BrCa [29–32]. In contrast, it is well accepted that the abundance of functional infiltrating cytotoxic T cells correlates with good prognosis in cancer patients [33, 34].

All this evidence indicates a complex relationship between different cell types, both locally and systemically, which requires further investigation. Here, we evaluated the systemic immune interactions that may modulate breast cancer development in obesity, with special emphasis on the cellular subpopulations that may promote a sustained immunosuppressive milieu characteristic of an established tumor.

## Materials and methods

### Mice

Experiments were performed with 4-week-old female C57BL/6 mice. All animals were bred and maintained in the animal facility of the Instituto de Investigaciones Biomédicas (IIB, UNAM, México), in specific pathogen-free conditions, according to the ethical guidelines. All procedures were approved by the Comité para el Cuidado y Uso de Animales de Laboratorio (CICUAL) of the Institute with protocol #228, that describe) methods of sacrifice (2) methods of anesthesia and/or analgesia, and (2) efforts to alleviate suffering.

E0771 murine breast cancer cell line was kindly provided by Dr. Kolonin (University of Texas, Health Science Center in Houston) [35].

### Animal model of obesity

Four weeks-old female mice were divided into two groups. Obese and lean mice were fed with 60% fat (high fat diet, HFD) and 10% fat diet (normal fat diet, NFD), respectively for 15 weeks (TestDiet^®^) and mice weight was monitored weekly. At week 12, blood samples were taken to test blood glucose levels after 12 h of fasting and the percentage of blood leukocytes was determined by flow cytometry.

### Breast cancer tumor model

Tumor implantation was performed as previously described. Briefly, 3x10^5^ tumor cells were resuspended in 1:1 PBS: Matrigel High Concentration (Corning) and subcutaneously injected in the fourth fat pad of the mammary gland [35, 36]. Control mice were also administered with saline solution. Three weeks after tumor implantation, mice were sacrificed, tumors were sized, and organs were processed and analyzed by flow cytometry.

### Processing of draining and peripheral lymph nodes and spleen

Lymph nodes and spleens were cut into small pieces and digested with 5 mg/mL collagenase IV (Thermo Fisher) and 25 U/mL of DNase (Roche) in RPMI 1640 5% FBS, for 30 minutes at 37°C. Then, tissues were mechanically disaggregated and filtered through a 100 μm nylon mesh and washed with PBS 1X. Erythrocytes were lysed with ACK buffer for 3 minutes and washed with PBS 1X. Isolated cells were washed and resuspended in PBS and maintained at 4°C until phenotypic analysis by flow cytometry.

### Tumor cell preparation

Tumors were weighed and measured to determine their volume ($v=\frac{1}{2}(LxW^{2})$) [37], subsequently incubated for 30 minutes at 37°C with RPMI 1640 (Thermo Scientific) supplemented with 5% FBS (BioWest), 5 mg/mL collagenase IV (Thermo Scientific), 25 U/mL DNase (Roche), and mechanically disaggregated. The resulting cell suspension was filtered through a 100 μm cell strainer (Corning) and washed with PBS 1X. Erythrocytes were lysed with ACK buffer for 3 minutes and washed with PBS 1X. For lymphocyte isolation, cell suspensions were layered on standard Ficoll-Paque^™^ Plus (GE Healthcare) and then separated by density-gradient at 500 *g*. Isolated cells were washed and resuspended in PBS and kept at 4°C until phenotype analysis by flow cytometry.

### Preparation of adipose tissue for immune cell infiltrate analysis

After euthanasia, we obtained VAT from the abdominal cavity. The adipose tissue was washed with adipocyte buffer (AB) (0.14 M NaCl, 4.7 mM KCl, 2.5 mM CaCl_2_, 1.2 mM MgSO_4_, 1.2 mM KH_2_PO_4_, 1 mM sodium pyruvate, 0.2% BSA, 20 mM HEPES, and 1% Anti/Anti (Thermo Scientific), pH 7.4). VAT was incubated with RPMI 1640 supplemented with 1 mg/mL collagenase I (Thermo Scientific), 1.5% Bovine Serum Albumin (Sigma-Aldrich), 5 mM Glucose (Sigma-Aldrich), 120 mM NaCl, 50 mM KCl, 1 mM CaCl_2_, and 100 mM HEPES (Sigma-Aldrich) for 1 hour at 37°C at 250 rpm. Digested tissue was washed twice with AB and centrifuged at 1800 rpm for 5 minutes. Cells were resuspended in PBS and kept at 4°C until phenotype analysis by flow cytometry.

### Preparation of peripheral blood cells for immune cell analysis

After 12 weeks of HFD or NFD feeding, blood samples were collected by submandibular vein puncture in a heparinized tube. Erythrocytes were lysed with ACK for 3 minutes, washed with PBS 1X, and centrifuged at 1800 rpm for 5 minutes. Cells were resuspended in PBS and maintained at 4°C until phenotype analysis by flow cytometry.

### Flow cytometry analysis

For flow cytometry analysis of lymph nodes (LN), spleen (SP), tumors, and AT, the following antibodies and buffers were used: CD3-PE, CD4-BV510, CD8-PeCy7, F4/80-APC, MHCII-AF488, CD206-BV605 CCR8-BV421, CD39-PeCy7, NRP1-PE and PD1-BV711 from Biolegend; CD25-PeCy5.5 from Thermo Scientific and CD11b-VF450 from Tonbo. For intracellular analysis, we used anti-Foxp3-APC (Tonbo) and Helios-FITC (Biolegend), using the Foxp3/Transcription Factor Staining Buffer Kit (Tonbo) for fixation and permeabilization of the cells.

All cells were initially stained with viability dye Zombie-NIR (Biolegend) and samples were acquired in an Attune NxT^®^ Acoustic Focusing Cytometer (Thermo Scientific) and flow cytometry data was analyzed using FlowJo 10.8.1 (Tree Star).

### Statistical analysis

Data are presented as mean ± SEM. The statistical analysis was performed with PRISM 5 software (GraphPad Software). The Kolmogorov–Smirnov test was used to evaluate the distribution of data sets. Statistical differences were analyzed by unpaired Student *t*-test, one-way ANOVA, with Bonferroni and Dunn´s post hoc test. P values <0.05 were considered as statistically significant. P values >0.05 and <0.1 were considered as trends.

## Results

### Decreased regulatory T cells and increased monocytes are observed in peripheral blood of obese mice

**Fig 1:**
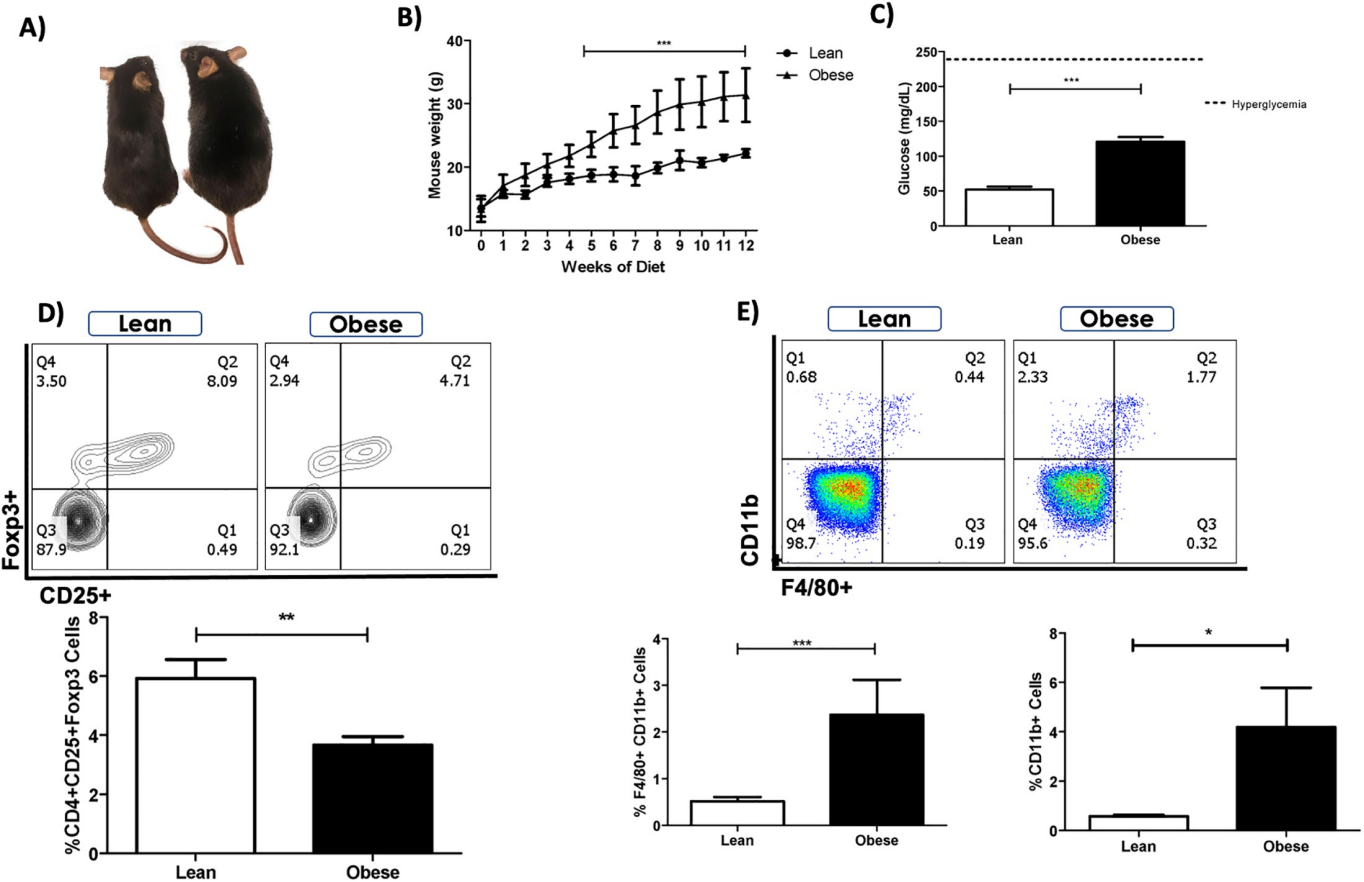
Decreased regulatory T cells and increased monocytes are observed in peripheral blood of obese mice. **A)** Representative images of lean (left) and obese (right) mice fed with NFD and HFD diet, respectively. **B)** Body weight of mice fed with NFD (circles) or HFD (triangles) was measured weekly for 12 weeks. **C)** At this time, blood glucose levels were measured in lean (white bar) and obese (dark bar) mice after a 12-hour fasting. Hyperglycemia is indicated with a dotted line. **D)** Representative **contour** plots (upper panels) and frequencies (lower panel) of CD4^+^ CD25^+^ Foxp3^+^ regulatory T cells found in peripheral blood of lean (left plot, white bar) and obese (right plot, dark bar) mice analyzed by flow cytometry after 12 weeks of diet intake. Quadrants were established based on the FMO values obtained for each marker **E)** Representative dot plots (upper panels) and percentages (lower panels) of CD11b^+^ F4/80^+^ monocytes and CD11b^+^ cells found in peripheral blood of lean (left plot, white bars) and obese (right plot, dark bars) mice at 12 weeks of diet intake. Data are expressed as Mean ± SEM of three independent experiments **(total of n = 12 mice)**. Statistical significance was determined by two-way ANOVA and paired two-tailed Student’s *t*-test. *p≤0.05, **p≤0.001, ***p≤0.0001.

Four-week-old C57BL/6 female mice were fed with either HFD or NFD, for twelve weeks (Fig 1). Body weight from both mice groups was measured weekly, showing a significant increase in the obese group compared to the control group, from the 5th to the 12th week of diet intake (Fig 1B). Glucose levels from HFD mice were significantly increased at 12 weeks compared to NFD mice (Fig 1C), not reaching the hyperglycemia reported in diabetic mice [38]. As previously reported, obesity resulted in a significant decrease in the percentage of circulating CD4^+^CD25^+^Foxp3^+^ regulatory T cells (Fig 1D) but a decreased proportion of peripheral blood monocytes (F4/80^+^ CD11b^+^ and CD11b^+^) (Fig 1E) compared to lean controls [39, 40].

### Obesity promotes tumor growth in a breast cancer mouse model

**Fig 2:**
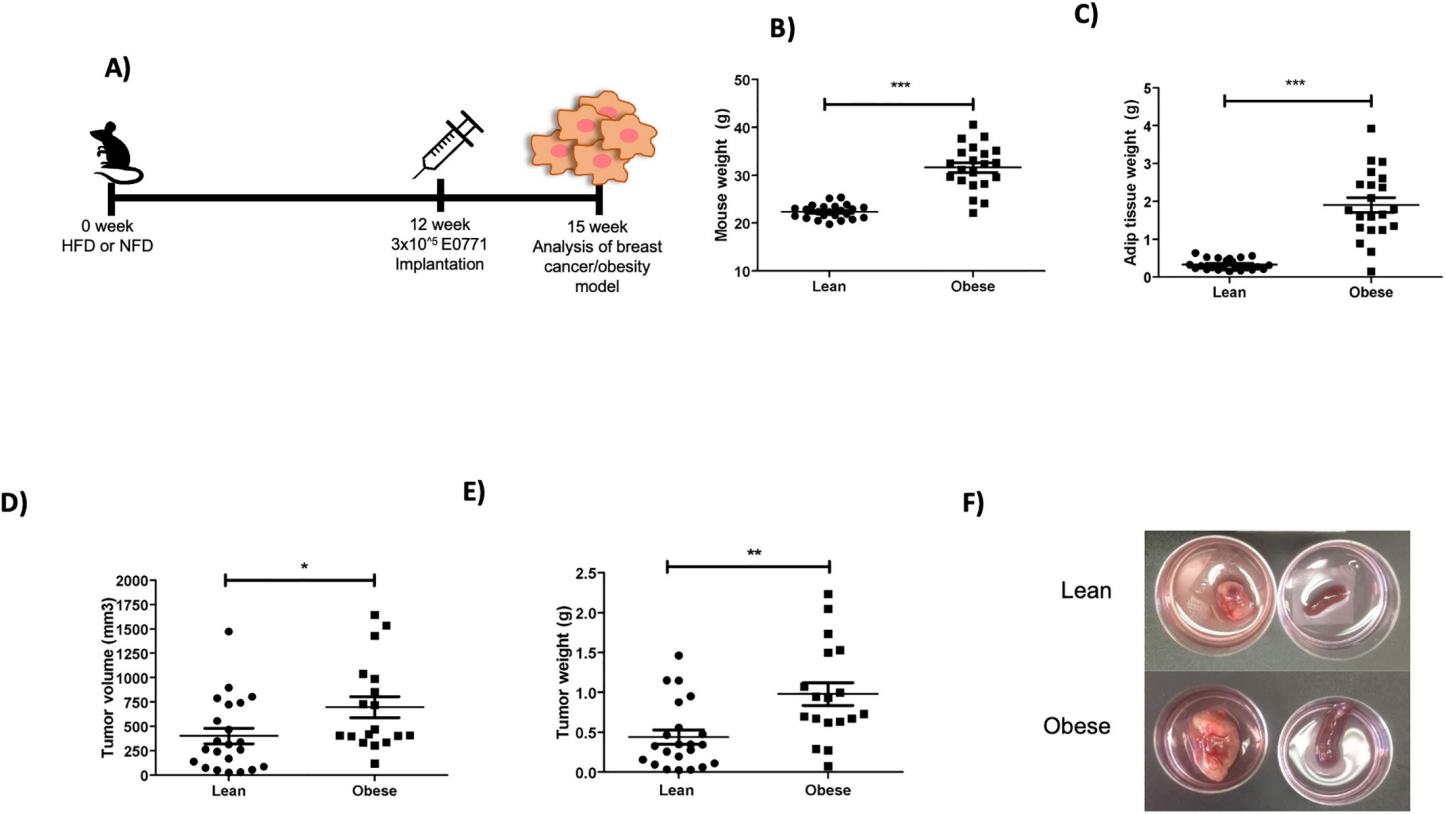
Increased tumor growth in mice fed with HFD. **A)** Schematic diagram of the obesity-breast cancer model used. Mice were fed for 12 weeks with HFD or NFD and then were implanted with E0771 cells. Three weeks after implantation, tumors and adipose tissue were analyzed **B)** Mouse weight, **C)** adipose tissue weight **D)** tumor volume, and **E)** tumor weight, were measured in lean (circles) and obese (squares) mice 3 weeks after tumor implantation. **F)** Representative images of tumors (left) and spleens (right) from lean (top) and obese (bottom) mice taken 3 weeks after tumor implantation. Data are expressed as Mean ± SEM of four independent experiments. Statistical significance was determined by paired two-tailed Student’s *t*-test. *p≤0.05, **p≤0.001, ***p≤0.0001.

We used the tumoral cell line E0771, to analyze the effect of obesity on the immune response to breast cancer. For this, cells were implanted in the mammary fat pad of obese or non-obese mice and allowed tumor growth for three weeks (Fig 2A). At the endpoint, mice, tumors, and adipose tissue were weighed and tumor volume calculated. As expected, HFD resulted in a significant increase in mouse and VAT weight compared to lean mice (Fig 2B and 2C) [35]. VAT was reduced in HFD tumor bearing mice (S2 Fig), possibly as a result of enhanced fatty acid consumption by the growing tumor. Interestingly, tumor volume and weight were significantly increased in obese mice in comparison with lean mice (Fig 2D and 2F). The same trend was observed when we analyzed mouse weight (excluding the tumor) versus tumor volume (S1 Fig) showing that tumor progression is promoted by obesity, as previously reported [41], indicating a restrained anti-tumoral immune response.

### Intratumoral regulatory T cells are not affected by obesity

**Fig 3:**
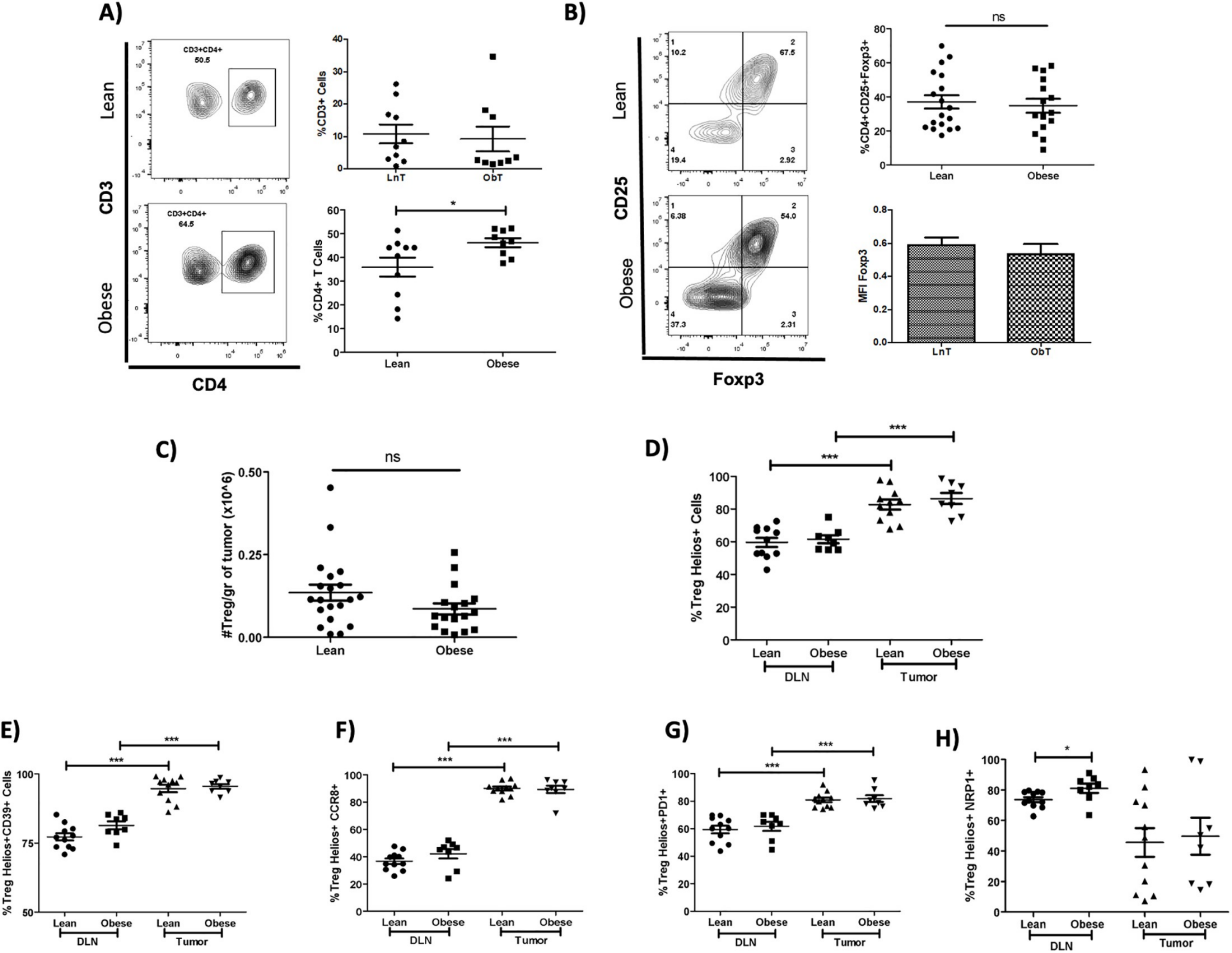
High fat diet does not alter the frequency, number, or phenotype of intratumoral regulatory T cells. Representative dot plots (left) and frequencies (right) of tumor infiltrating **A)** CD4+ T cells and **B)** CD4^+^ CD25^+^ Foxp3^+^ regulatory T cells in lean (upper plot, circles) and obese (lower plot, squares) mice 3 weeks after tumor implantation. **C)** Numbers of intratumoral regulatory T cells per gram of tumor. Frequencies of **D)** Helios^+^, **E)** Helios^+^ CD39^+^, **F)** Helios^+^ CCR8^+^, **G)** Helios^+^ PD-1^+^, and **H)** Helios^+^ NRP1^+^ intratumoral and DLN Tregs from lean (circles) and obese (squares) mice. Data are expressed as Mean ± SEM of three independent experiments. Statistical significance was determined by two-tailed Student’s *t*-test and one-way ANOVA. * p≤0.05, ***p≤0.0001.

It has been shown that increased intratumoral Tregs correlates with cancer progression in several models, including colorectal cancer, melanoma, and breast cancer [42, 43]. Therefore, we decided to evaluate the impact of obesity on tumor immune cell infiltration. A significant increase in the percentage of intratumoral CD4^+^ T cells was observed in obese mice compared to lean mice (Fig 3A; lower panel), despite there being no changes in the percentages of CD3^+^ cells (Fig 3A; upper panel). However, when we analyzed CD4^+^ T cell subpopulations, we found no significant differences in the percentages (Fig 3B) and numbers (per gram of tumor) (Fig 3C) of intratumoral Tregs in obese mice, compared to lean mice. This is in accordance with the lack of correlation between CD4+CD25+ Foxp3+ infiltrating Tregs and the tumor volume both in lean and obese mice (S3 Fig). In addition, Foxp3 expression in intratumoral Tregs from obese mice was not significantly different from that of lean mice (Fig 3B, lower panel). As expected, the majority of Tregs infiltrating breast cancer tumors are of thymic origin [44], which characteristically express Helios (a transcription factor associated with tTregs that promotes Foxp3 expression and stability). Accordingly, we found an increase in Helios^+^ Tregs infiltrating the tumor, compared to draining lymph nodes, although this effect was not significantly different between lean and obese mice (Fig 3D).

Intratumoral Tregs from BrCa patients have been shown to display a distinctive phenotype in comparison with peripheral Tregs, characterized by the upregulation of CD39, CCR8, and PD-1 [29]. Therefore, we next evaluated these markers in both intratumoral and draining lymph node (DLN) Tregs from either lean or obese mice. As shown in Fig 3E and 3G, the percentage of CD39^+^, CCR8^+^, and PD-1^+^ cells within the Helios^+^ Treg subpopulation were significantly increased in intratumoral Tregs compared to DLN Tregs. However, the upregulation of these markers, typically associated with an increased suppressive function [45], was similarly observed in obese and lean mice, indicating that this phenotype is not affected by obesity. In contrast, the frequency of Neuropilin-1^+^ (NRP1) Helios^+^ Tregs was not significantly increased within the tumor compared to DLN in either obese or lean mice (Fig 3H).

Our results confirm that intratumoral regulatory T cells may have a more suppressive function, which may be imprinted by the tumor, although this phenotype does not appear to be modified by obesity.

### Decreased frequency of CD8^+^ infiltrating T cells and altered macrophage polarization in tumors from obese mice

**Fig 4:**
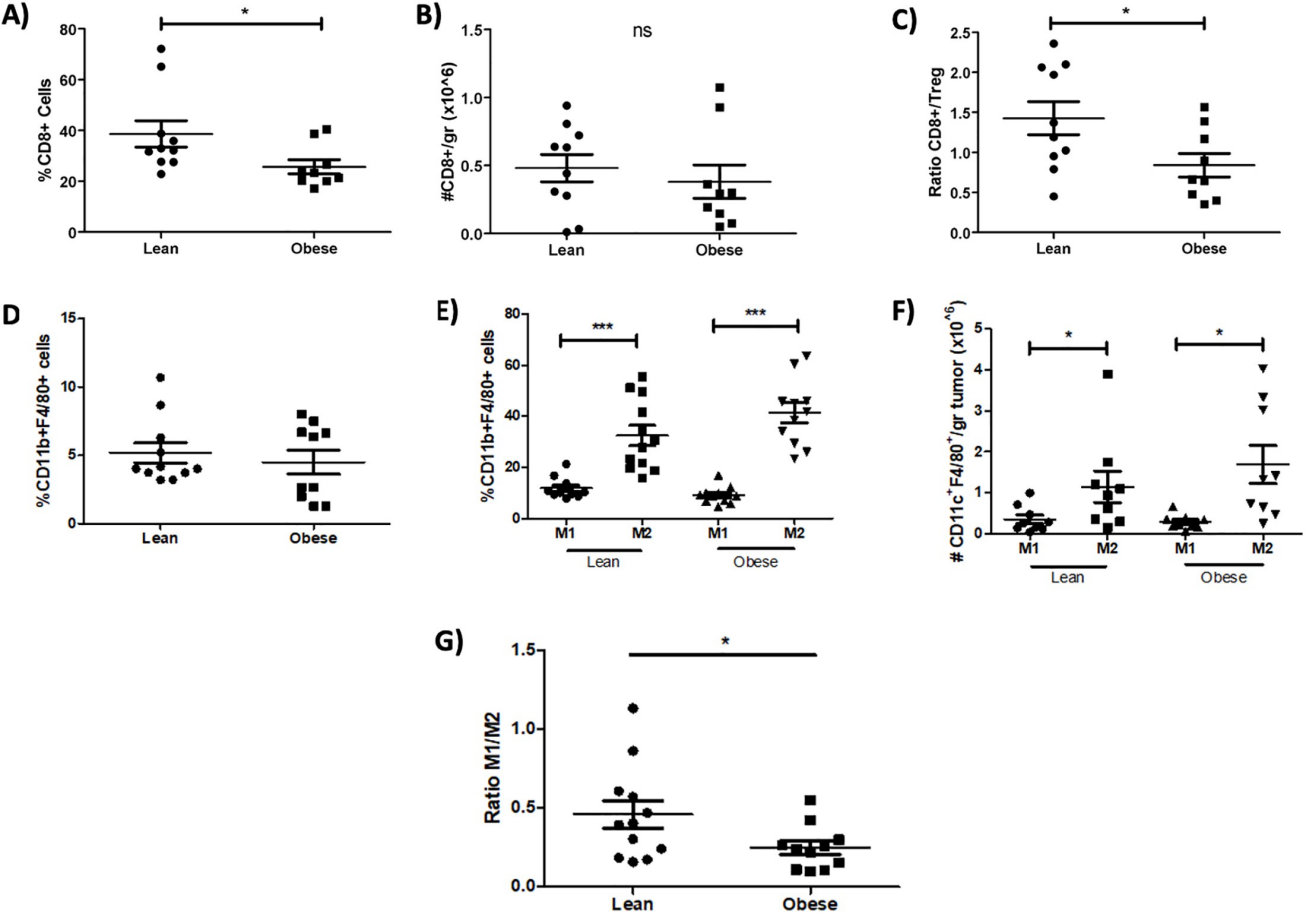
Decreased frequency of CD8+ cells and M1/M2 macrophage ratio in obese mice might indicate an impaired anti-tumoral reponse. **A)** Percentages and **B)** total numbers of tumor infiltrating CD8+ T cells from lean and obese mice. **C)** CD8+/Treg ratio found in tumors. **D)** Percentages of intratumoral CD11b^+^ F4/80^+^ macrophages. **E)** Percentages and **F)** total numbers of MHCII^+^ M1 and CD206^+^ M2 macrophages found in tumors of lean and obese mice. **G)** M1/M2 macrophage ratio identified in tumors of lean and obese mice. Data are expressed as Mean ± SEM of three independent experiments. Statistical significance was determined by paired two-tailed Student’s *t*-test and one-way ANOVA. *p≤0.05, **p≤0.01, ***p≤0.0001.

As obesity does not appear to have a clear impact on the intratumoral Tregs subpopulation, we next evaluated other immune populations that are important in regulating the balance between an effective anti-tumoral response and tumor escape. In this context, it is known that CD8+ T lymphocytes [46, 47] play a key role in anti-tumoral responses and promote tumor progression [48, 49]. As shown in Fig 4, a diminished percentage (Fig 4A) and slight decrease in the number of CD8^+^ T cells per gram of tumor (Fig 4B) in obese mice compared to lean mice. Notably, the ratio between CD8^+^ T cells and Tregs (CD8^+^/Treg) infiltrating the tumor was significantly decreased in obese mice compared to lean mice (Fig 4C), providing further evidence that the anti-tumoral immune response may be compromised in obesity, favoring tumor escape.

Myeloid cells have been shown to modulate the TME favoring tumor growth and metastasis [50]. Specifically, M1-like macrophages are known to be more predominant in the early stages, while M2-like polarized macrophages are associated with late stages of tumor development. Obesity has been shown to alter the myeloid subpopulations both in peripheral blood and AT. Therefore, we hypothesized that the profile of macrophages infiltrating the tumor might be modulated by this low-grade inflammatory process. Although the percentages of CD11b^+^ F4/80^+^ present within the tumor were not significantly different between obese and lean mice (Fig 4D), we observed a higher percentage (Fig 4E) and number (Fig 4F) of the M2 versus the M1 macrophages, as it has been reported in other tumors [51, 52]. Accordingly, the M1/M2 ratio was significantly decreased (Fig 4G), indicating that obesity may promote an enhanced immunosuppressive milieu that has been described to favor tumor growth [53]. However, we found no significant correlation between the M1/M2 ratio and tumor volume in obese mice (S4 Fig), suggesting that obesity may affect tumor development through other mechanisms besides the alteration of the immune microenvironment.

In summary, our data show that obesity impairs the anti-tumoral immune response, which is associated with a reduced CD8^+^ T cell infiltration and an alternative activation of macrophages.

### Effect of tumor growth on peripheral lymphocyte subpopulations

**Fig 5:**
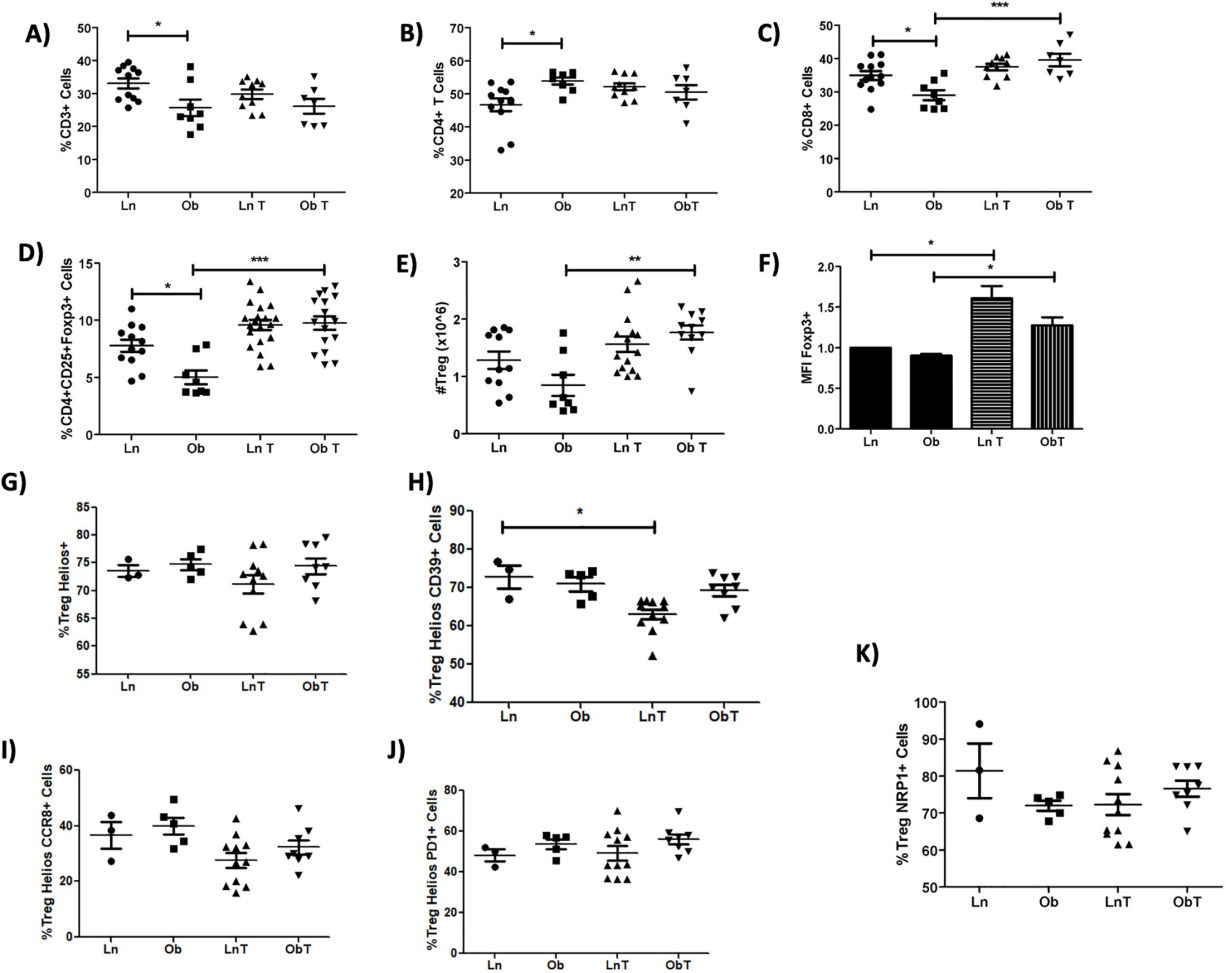
Tumor growth increases the frequency and number of regulatory T cells in the spleen of obese mice without affecting their phenotype. **A)** Percentages of CD3^+^ **B)** CD4^+^, **C)** CD8^+^, **D)** regulatory T cells, and **E)** total numbers of Tregs found in the spleen of tumor-bearing or control lean and obese mice. **F)** Foxp3 expression in splenic regulatory T cells. Percentages of **G)** Helios^+^, **H)** Helios^+^CD39^+^, **I)** Helios^+^CCR8^+^, **J)** Helios^+^PD-1^+^, and **K)** Helios^+^NRP1^+^ regulatory T cells in the spleen of tumor-bearing or control lean and obese mice. Data are expressed as Mean ± SEM of three independent experiments. Statistical significance was determined by one-way ANOVA. *p≤0.05, **p≤0.01, ***p≤0.0001.

There is growing evidence that both obesity and tumor development have an impact in immune cells, affecting systemic immune responses [26, 54]. Therefore, we next evaluated whether alterations in peripheral immune subsets could be involved in the increased tumor growth observed in obese versus lean mice. As previously reported [39, 55], obesity led to a decreased frequency in CD3^+^ and regulatory T cells (Fig 5A and 5D) and an increase in the percentage of CD4^+^ T cells (Fig 5B). In contrast to previous studies, we found a decreased percentage of peripheral CD8^+^ T cells in obese mice. Interestingly, tumor development resulted in an increased percentage of CD8^+^ T cells and regulatory T cells in the spleen in both lean and obese mice (Fig 5C and 5D). Total Treg cells were also increased in numbers (Fig 5E) and expressed higher levels of Foxp3 in comparison with Tregs from mice without tumors (Fig 5F). Further analysis of splenic Tregs from tumor-bearing mice (Fig 5G and 5K) did not show an enrichment of the characteristic tumor-infiltrating Treg subpopulations, which display a highly suppressive phenotype (Fig 3D and 3H). In contrast, no differences were observed in splenic macrophages from obese compared to lean mice, with or without tumors (data shown).

### Alteration of leukocyte infiltration in cancer-associated adipose tissue in obese mice

**Fig 6:**
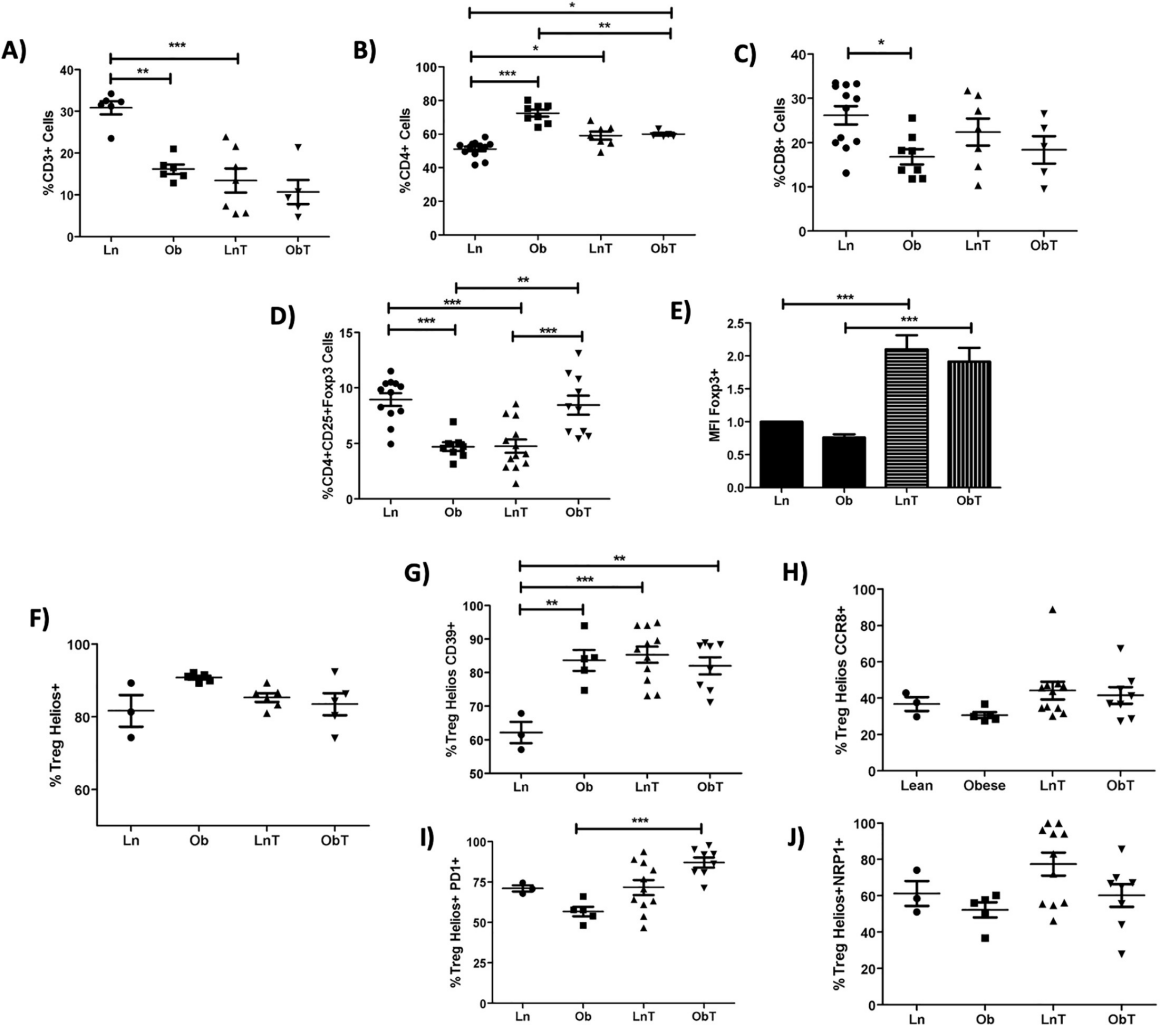
Tumor growth alters VAT T cell subpopulations **A)** Percentages of CD3^+^ **B)** CD4^+^, and **C)** CD8^+^ lymphocytes present in VAT of tumor-bearing or control lean and obese mice. **D)** Percentage and expression of Foxp3 **E)** in VAT Tregs from tumor-bearing or control lean and obese mice. **F-J)** Phenotypic analysis of Treg markers. Percentage of **F)** Helios^+^ **G)** Helios^+^CD39^+^, **H)** Helios^+^CCR8^+^, **I)** Helios^+^PD-1^+^, and **J)** Helios^+^NRP1^+^ VAT Tregs from tumor-bearing or control lean and obese mice. Data are expressed as Mean ± SEM of three independent experiments. Statistical significance was determined by one-way ANOVA. *p≤0.05, **p≤0.01, ***p≤0.0001.

Previous studies have described that CAAs (cancer-associated adipocytes) contribute to the recruitment of several immune subpopulations to the TME and aid other immune cells through the production of several metabolites and adipokines, which provide the metabolic conditions required for their differentiation into tumor promoting cells [56]. Reciprocally, tumor cells might potentially modulate immune cell populations infiltrating the AT, leading to systemic alterations of the immune and/or metabolic homeostasis. When we analyzed lymphocyte AT subpopulations in obese mice, we found that CD3^+^ cells and Tregs were decreased (Fig 6A and 6D), while CD4^+^ T cells were increased (Fig 6B), as previously reported [39, 55]. Unexpectedly, contrary to previous studies [57], we found a decreased percentage in CD8^+^ T cells in AT from obese mice (Fig 6C). Interestingly, tumor development by itself was able to significantly decrease the percentage of VAT Tregs, to the same extent as that caused by obesity. In contrast, the presence of tumors in obese mice resulted in a significant increase of VAT Tregs, apparently counteracting the pro-inflammatory effect of obesity (Fig 6D). Notably, VAT-Tregs from tumor bearing mice (both obese and lean) overexpressed Foxp3 (Fig 6E), correlating with the enhanced suppressive function of tumor-associated Tregs [45, 58]. Moreover, Helios ^+^ VAT Tregs (Fig 6F) from obese mice and tumor-bearing mice were enriched in CD39^+^ and PD-1^+^ cells, while no differences were observed in the expression of neither NRP-1^+^ nor CCR8^+^ Tregs (Fig 6G and 6J).

**Fig 7:**
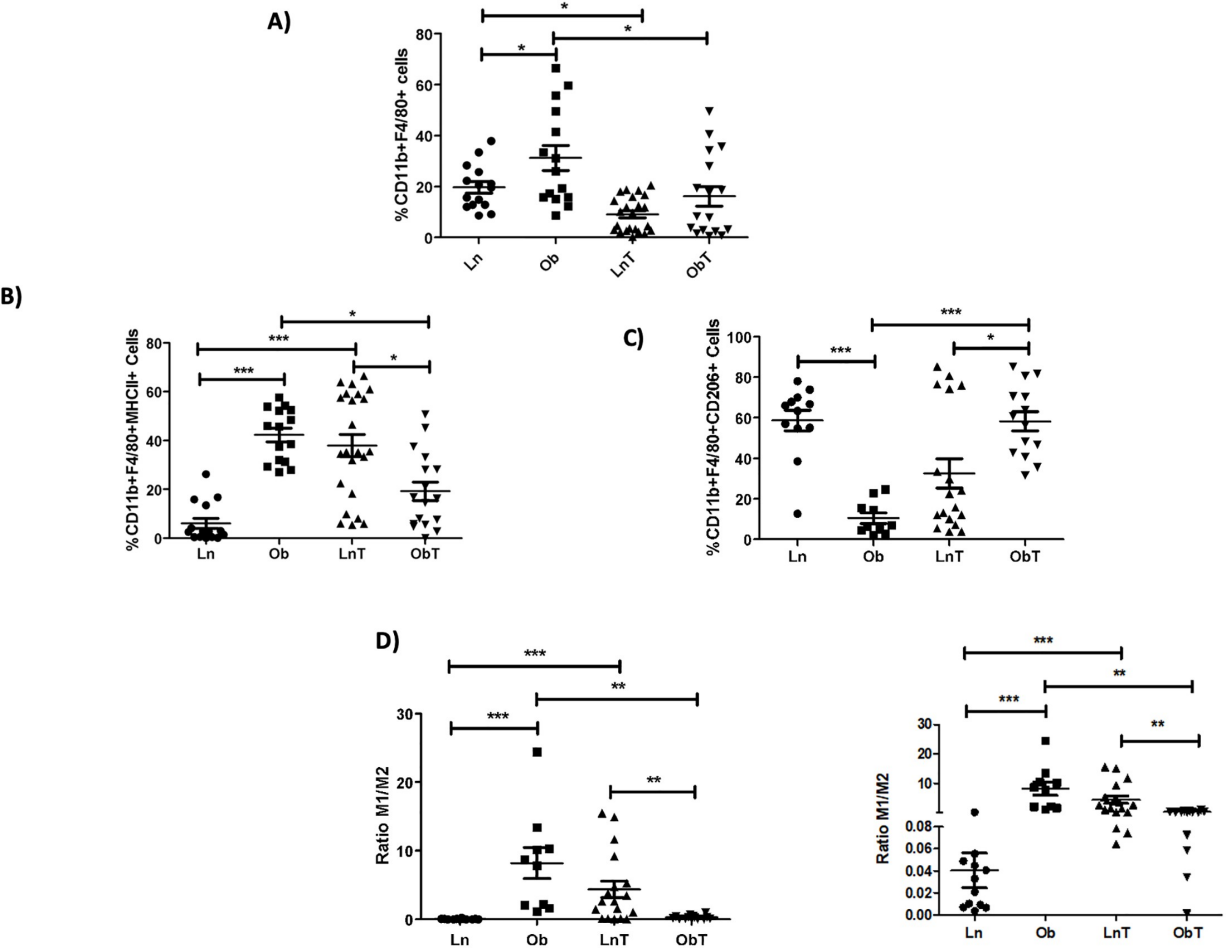
Tumor development alters the percentage and M1/M2 phenotype of VAT macrophages in obese mice. Percentages of **A)** CD11b^+^ F4/80^+^ **B)** MHCII^+^ M1, and **C)** CD206^+^ M2 VAT macrophages and **D)** M1/M2 ratio found in VAT from tumor-bearing or control lean and obese mice. Data are expressed as Mean ± SEM of three to four independent experiments. Statistical significance was determined by one-way ANOVA. *p≤0.05, **p≤0.01, ***p≤0.0001.

We next evaluated the effect of obesity and tumor development in AT macrophage subpopulations. As previously reported [59], we found a significant increase in the percentage of infiltrated macrophages in the AT of obese mice, while these cells were significantly decreased in the presence of tumors, both in lean and obese mice (Fig 7A). As previously described [60, 61], we observed an increment in the M1 (MHCII^+^) macrophages and a decrease in M2 (CD206^+^) macrophages in VAT from obese mice compared to lean mice (Fig 7B and 7C). Interestingly, tumor development in obese mice decreased the characteristic high M1/M2 ratio of fat AT, while promoting the increase of this ratio in lean mice (Fig 7D).

## Discussion

There is growing evidence showing the association between obesity and cancer development; however, the potential contribution of the systemic immune dysregulation caused by obesity remains controversial. Here, we used an experimental mouse model of HFD-induced obesity in C57/BL6 mice [62] to investigate whether local and/or systemic immune cell interactions may be involved in breast cancer progression. In this model, gradual accumulation of adipose tissue in the mesenteric depot begins at an early age and is accompanied by the metabolic alterations characteristic of obesity, after 16 weeks of HFD intake. In our experiments, we evaluated blood glucose levels to confirm that all mice included in the study were normoglycemic (<240 mg/dl), as previous reports have shown that obesity can be accompanied with diabetes development [38].

The effect of obesity in peripheral immune cell subpopulations has been extensively documented [63]. Our data show that obesity results in an increase in circulating myeloid cells, specifically the CD11b^+^ F4/80^+^ monocyte subpopulation, similarly to previous reports [64]. This increase correlates with the enhanced homing capacity and inflammatory phenotype observed in VAT macrophages from diet-induced obese mice [40].

In addition, we observed a decrease in the percentage of circulating CD4^+^CD25^+^ Foxp3^+^ Treg and an increase in CD4^+^ T cells, which correlates with the immune dysregulation previously described in obese mice and humans [65, 66]. In this context, obesity was has been shown to affect Treg mobilization by altering their phenotypic features, such as homing patterns of VAT peripheral Tregs as seen by the enhanced expression of CCR7 in splenic Tregs and their reduced VAT infiltration [39], which may establish an inflammatory state, implicated in the intratumoral infiltration in HFD-fed animals, therefore promoting tumor growth. Interestingly, the presence of tumors counteracted some of the immune alterations reported in obesity, such as the decreased percentage of Tregs [67–69], which further supports the systemic immunosuppression promoted by the tumor [70].

Several mechanisms have been proposed to explain the association of obesity with cancer development both in mouse models [71–75] and in cancer patients [76]. Among them, a systemic low-grade chronic inflammatory state that involves local and systemic alterations in immune cells and their mediators [77], affecting the recruitment, phenotype and function of specific subpopulations within the TME. In addition, increased expression of hs-CRP in peripheral blood was associated with increased risk of cancer development [78].

Specifically, Tregs play a key role in suppressing the anti-tumoral immune response mediated by NK and CD8^+^ T cells [79]. In this context, recent reports have clearly shown the effectiveness of PD-1/PD-L1 checkpoint blockade in preventing anti-tumoral cytotoxic CD8+ T cell exhaustion [80] and counteracting the suppressive function of infiltrating Tregs leading to improved response to cancer therapy increase disease free survival [81]. However, in our model, Tregs were not significantly increased in tumors from obese mice (Fig 3B and 3C), similarly to what was observed in a previous study in melanoma [82]. However, we found a significant decrease in the CD8^+^ T cell/Treg ratio, which is considered a poor prognosis parameter in breast cancer [83, 84], suggesting that obesity promotes tumor progression by impairing the local cytotoxic T cell response, rather than promoting the recruitment and/or expansion of intratumoral Tregs.

Recent studies have shown that phenotypical and functional features of intratumoral Tregs are biologically relevant for tumor progression [45, 85]. In addition to Foxp3, upregulated expression of Helios [86, 87], PD-1 [88], CD39 [89, 90], Neuropilin-1 [91] and CCR8 [29] have been strongly associated with impaired anti-tumoral responses in BrCa. Accordingly, intra-tumoral Helios^+^ Tregs were enriched in CCR8^+^ CD39^+^ PD-1^+^ subpopulations compared to DLN and splenic Tregs (Fig 3), both in obese and lean mice, further supporting the notion that tumor progression is associated with an enhanced immunosuppressive milieu [92] but suggests that obesity is not involved in the accumulation of intratumoral Tregs.

Growing evidence has shown that tumor growth is promoted by obesity through myeloid cell differentiation and polarization. In addition to inducing an increase in the frequency of circulating monocytes (Fig 1), obese AT contributes to the recruitment of macrophages into the TME through the production of IL-1β, CCL2, and CXCL12 [93] and the release of proinflammatory molecules like free-DNA from necrotic adipocytes [94]. Moreover, AT has been shown to be a major source of TAMs [95], which have a characteristic M2-like phenotype and impair cytotoxic anti-tumoral T cell responses, enhancing the immunosuppressive microenvironment [96]. Accordingly, we observed a lower M1/M2 ratio in obese mice tumors, which also has been correlated with poor prognosis in obese cancer patients [97].

In addition to M2s, MDSCs have also been described to have an important role in tumor progression. Interestingly, a recent study using our same cancer model reported that obesity-associated MDSCs promote apoptosis of tumor infiltrating CD8+ T cells and are associated with resistance to immunotherapy [98], demonstrating the complexity of the immune cell interactions taking place within the tumor, which is currently the focus for the development of new cancer immunotherapy treatments.

Obesity also affects the local and systemic production of immune cell mediators and adipokines. In this context, obese AT shows an altered phenotype and function, which includes the overproduction of leptin and the production of pro-inflammatory cytokines, including IL-6 and TNF-α, by adipocytes [99]. Leptin abundance has been associated with enhanced CD8^+^ T and CD4^+^ Th1 cell proliferation, increased M1/M2 ratio [100, 101] within the fat AT and reduced proliferation and maintenance of VAT Tregs [10]. In agreement, we found a significant decrease in the percentage of CD4^+^ CD25^+^ Foxp3+ Tregs in AT of HFD-treated mice (Fig 6D), as previously reported [102].

Concomitant with the effect exerted by obesity on the intratumoral microenvironment, we hypothesized that the tumor could also induce significant changes in the immune cell infiltrate within the AT, which might be biologically relevant. Notably, while VAT Tregs were significantly increased in obese mice bearing tumors, M1/M2 ratio was diminished (Fig 7D), suggesting that the tumor favors an anti-inflammatory milieu within AT. Conversely, tumor growth in lean mice led to the opposite effect in the proportion of Tregs and M1/M2 macrophages, resembling the inflammatory profile observed in fat adipose tissue.

**Fig 8:**
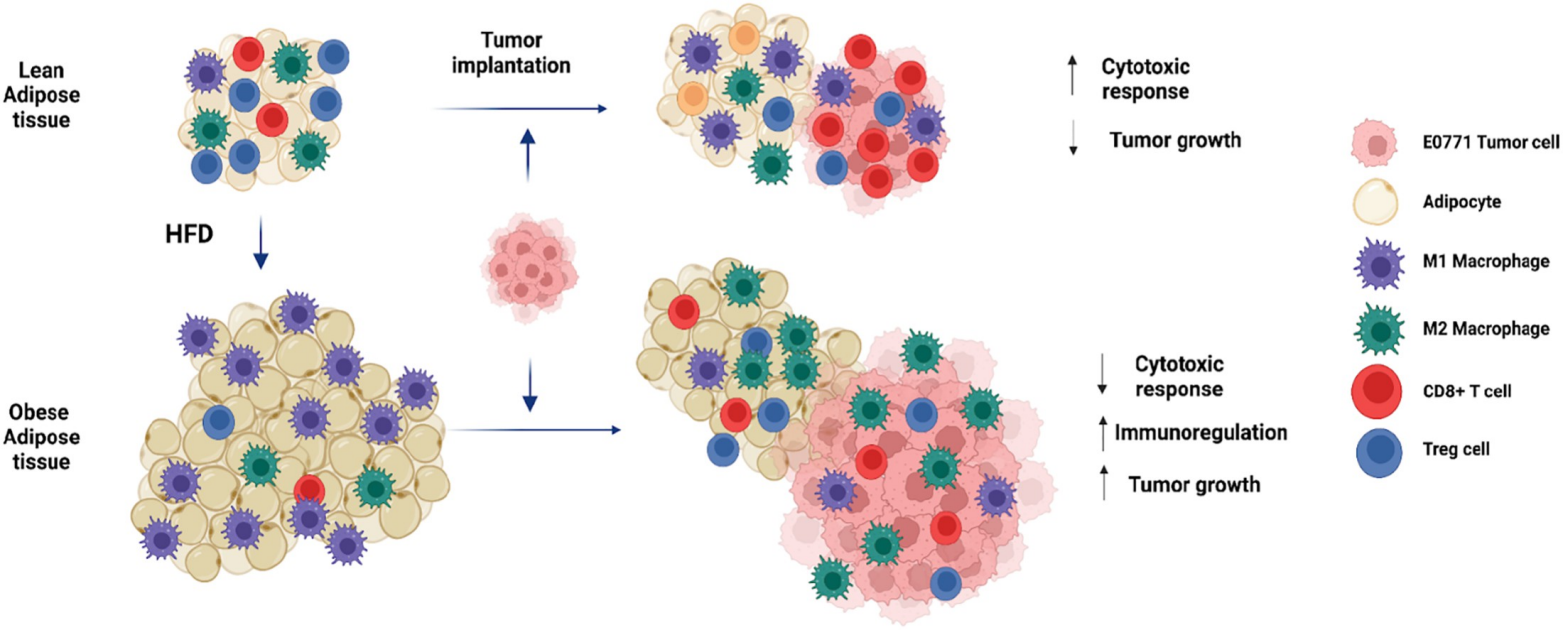
Schematic representation of the crosstalk communication between AT and the TME, through the modulation of local and systemic immune responses, which generates a systemic immunosuppressive macroenvironment that promotes tumor growth.

Altogether, our results indicate that obesity promotes BrCa tumor progression by enhancing macrophage recruitment, increasing M2 versus M1 macrophage differentiation and reducing anti-tumoral cytotoxic CD8^+^ T cell infiltration within the TME (Fig 8).

Our model also demonstrates the existence of a crosstalk communication between AT and the TME, through the modulation of local and systemic immune responses, which generates a systemic immunosuppressive macroenvironment that continues to support cancer progression.

In summary, our results uncover potential immune interactions established between the adipose tissue and the tumor, which may underlie the reported association between obesity and breast cancer progression.

## Supporting information

S1 Fig Positive correlation between mouse net weight and tumor volume. **A)** Mouse net weight and tumor volume were measured 3 weeks after tumor implantation. Data from 4 independent experiments.
(TIFF)

S2 Fig Tumor development leads to decreased visceral adipose tissue weight in obese mice but not in lean mice. Visceral adipose tissue was weighed after 3 weeks of the Bca implantation. Data are expressed as Mean ± SEM of three independent experiments. Statistical significance was determined by one-way ANOVA. *p≤0.05, **p≤0.01, ***p≤0.0001.
(TIFF)

S3 Fig Percentage of Tregs infiltrating the tumor is moderately correlated with tumoral volume. Correlation analysis at the 3rd week of implantation of E0771 Bca cells, between tumor volume and the percentage of CD4+CD25+FoxP3+ intratumoral Tregs Statistical significance was determined by two-tailed Student’s *t*-test and one-way ANOVA. Significant correlations were considered when r ≥0.9.
(TIFF)

S4 Fig Tumor growth correlates with the development of a suppressive intratumoral microenvironment in lean mice. Correlation between tumor volume and A) Percentage of CD11b+ F4/80+ MHC-II+ cells, B) Percentage of CD11b+ F4/80+ CD206+ cells and C) M1/M2 ratio versus tumor volume (mm^3^). Statistical significance was determined by two-tailed Student’s t-test and one-way ANOVA. Significant correlations were considered when r ≥0.9.
(TIFF)
