# Supplementary figures and images for: Obesity modulates the immune macroenvironment associated with breast cancer development
Source: PLoS One. 2022 Apr 26;17(4):e0266827. doi: 10.1371/journal.pone.0266827 (PMC9041840; doi:10.1371/journal.pone.0266827)

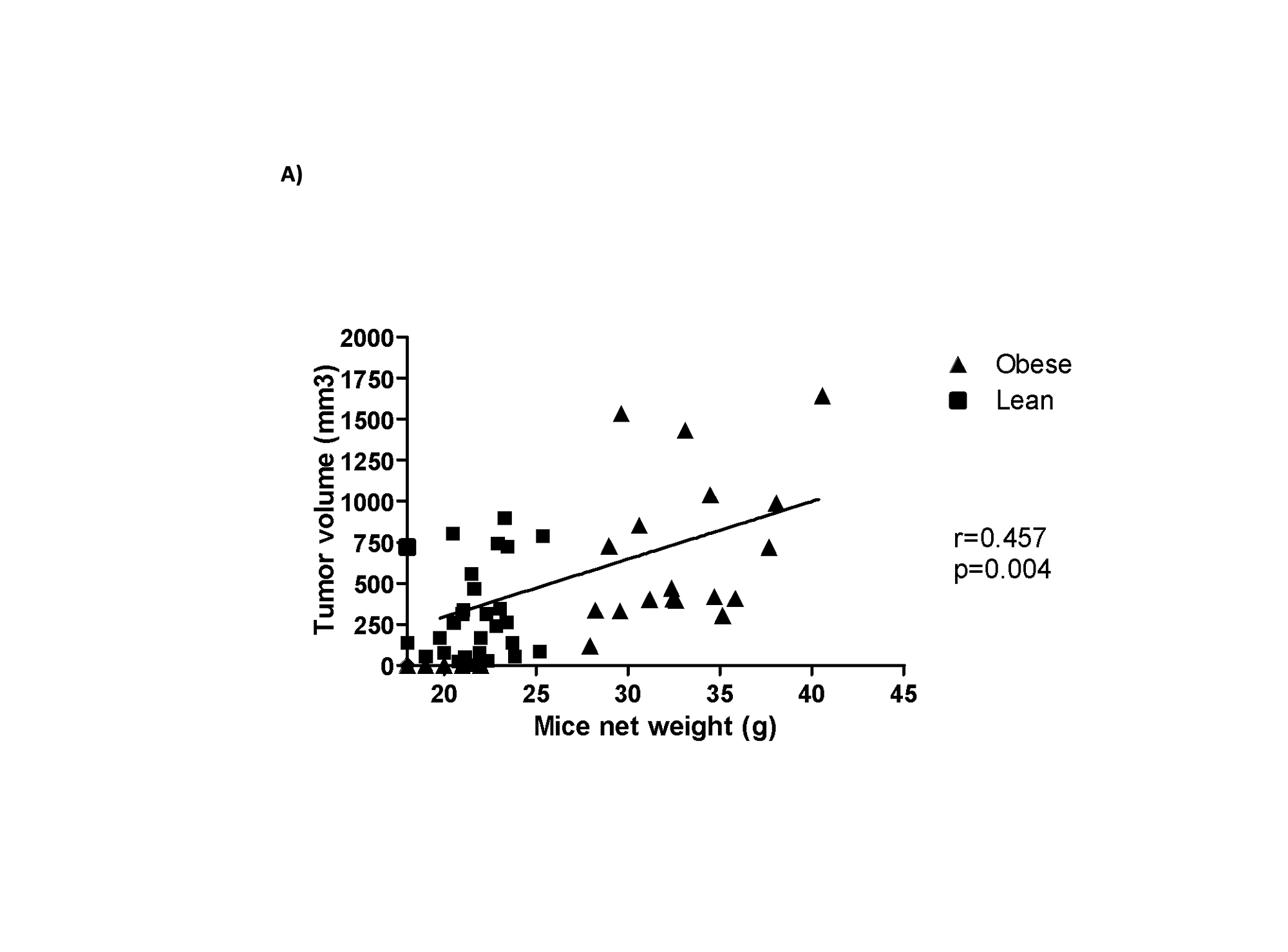

Supplement: S1 Fig — A) Mouse net weight and tumor volume were measured 3 weeks after tumor implantation. Data from 4 independent experiments. (TIFF) [file pone.0266827.s001.tiff]

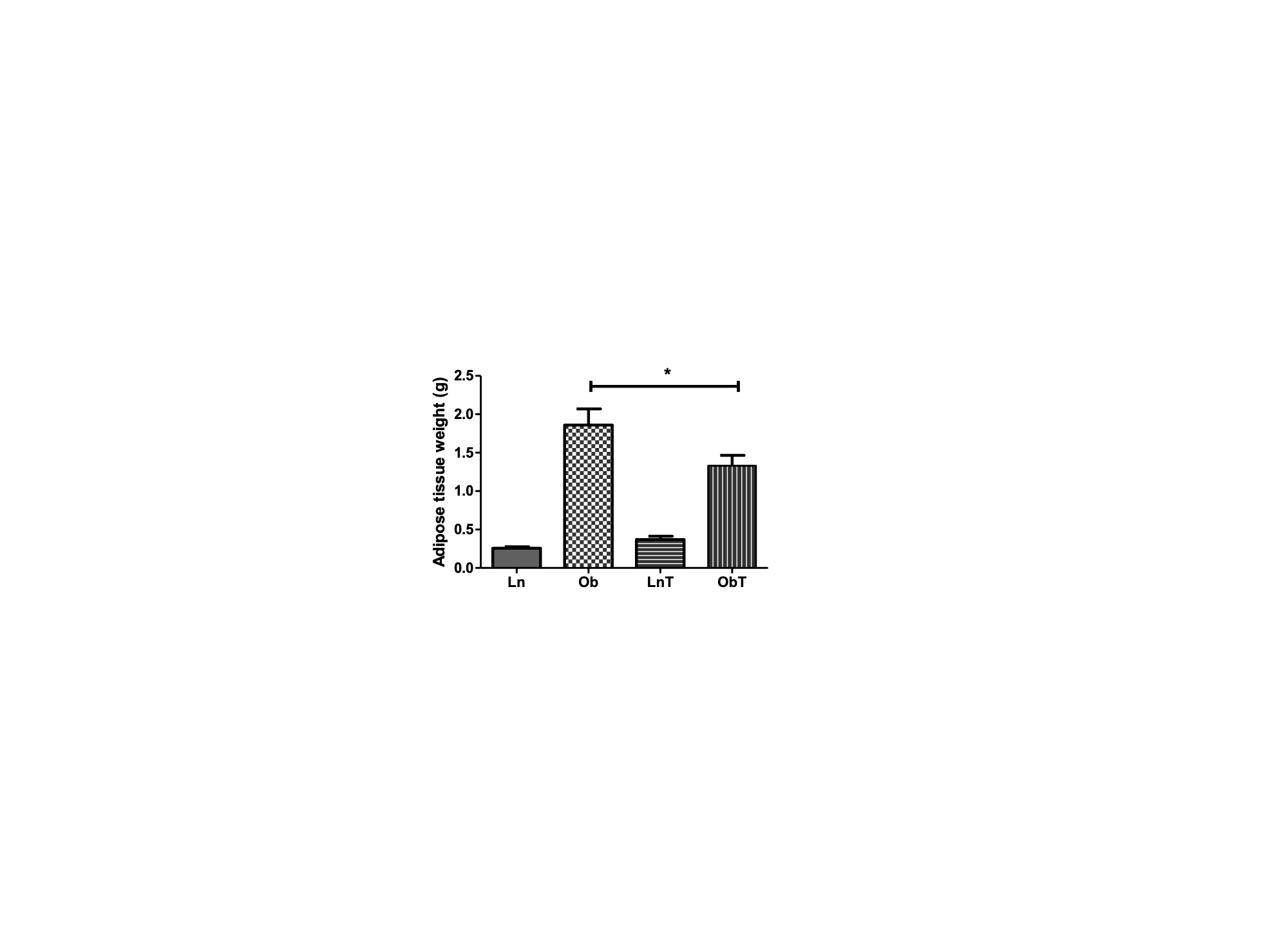

Supplement: S2 Fig — Visceral adipose tissue was weighed after 3 weeks of the Bca implantation. Data are expressed as Mean ± SEM of three independent experiments. Statistical significance was determined by one-way ANOVA. *p≤0.05, **p≤0.01, ***p≤0.0001. (TIFF) [file pone.0266827.s002.tiff]

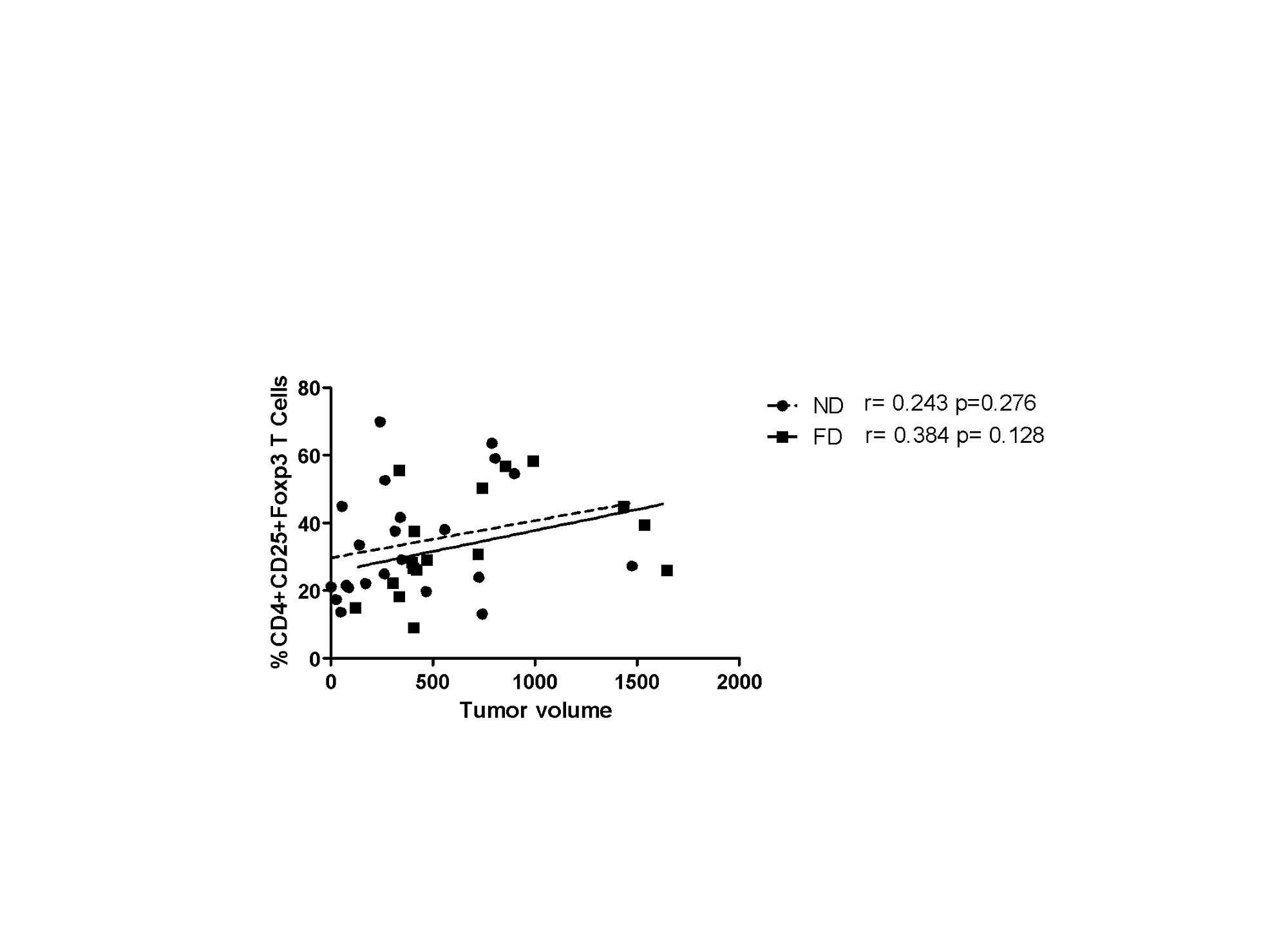

Supplement: S3 Fig — Correlation analysis at the 3rd week of implantation of E0771 Bca cells, between tumor volume and the percentage of CD4+CD25+FoxP3+ intratumoral Tregs Statistical significance was determined by two-tailed Student’s t-test and one-way ANOVA. Significant correlations were considered when r ≥0.9. (TIFF) [file pone.0266827.s003.tiff]

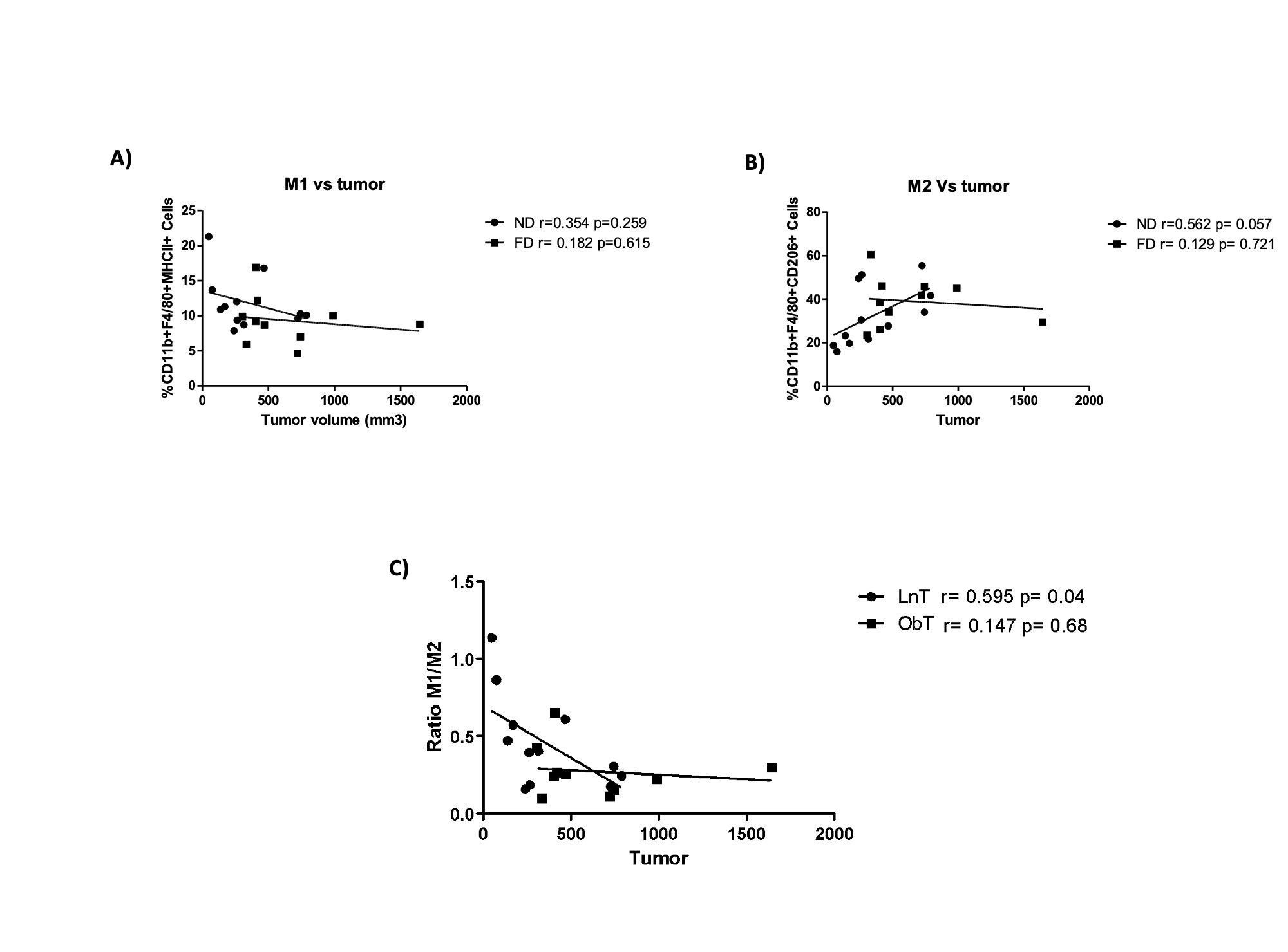

Supplement: S4 Fig — Correlation between tumor volume and A) Percentage of CD11b+ F4/80+ MHC-II+ cells, B) Percentage of CD11b+ F4/80+ CD206+ cells and C) M1/M2 ratio versus tumor volume (mm3). Statistical significance was determined by two-tailed Student’s t-test and one-way ANOVA. Significant correlations were considered when r ≥0.9. (TIFF) [file pone.0266827.s004.tiff]
